# Repurposing of the bisbenzimide antibiotic Ridinilazole as an antiviral compound against human cytomegalovirus

**DOI:** 10.1099/jgv.0.002205

**Published:** 2025-12-19

**Authors:** Hayden Pinto, Katie A. Latham, Steve Goodbourn, Adam A. Witney, Blair L. Strang

**Affiliations:** 1 Institute of Infection & Immunity, St George’s School of Health and Medical Sciences, City St George’s, University of London, London, UK

**Keywords:** bisbenzimide, compound, cytomegalovirus, drug, human, Ridinilazole

## Abstract

Novel antiviral drugs targeting DNA viruses are desirable. Previous studies from our laboratory and others have demonstrated that DNA binding bisbenzimide compounds are capable of inhibiting replication of diverse DNA viruses, such as a herpesvirus and prototype or pandemic potential poxviruses. To further the aforementioned studies, we sought to identify a bisbenzimide compound that had been successfully administered to humans and repurpose that compound as a broadly acting antiviral compound. We found that the previously described bisbenzimide antibiotic Ridinilazole was a potent inhibitor of human cytomegalovirus (HCMV) at sub-micromolar concentrations, with no obvious effect on cell viability. However, inhibition of virus replication by Ridinilazole was selective, as Ridinilazole had no obvious effect on the replication of another DNA virus (herpes simplex virus) or an RNA virus (influenza). Based upon our bioinformatic analysis of viral genome content, we propose that the number of putative Ridinilazole binding sites in a viral DNA genome is important for the antiviral action of Ridinilazole. Western blotting and electron microscopy revealed that Ridinilazole had no obvious effect on HCMV protein production, but did decrease the number of HCMV capsids in the cytoplasm. Overall, we identified an antibiotic compound previously used in humans that could be repurposed as an antiviral compound to efficaciously inhibit replication of HCMV.

## Introduction

Antiviral drugs to treat disease in humans are essential in many circumstances, including situations where vaccines are not available. This can be true of situations where previous drug discovery efforts have not been successful or where new viruses have emerged in a human population. Moreover, it is also desirable to identify novel antiviral compounds that target many viruses, as these compounds will be useful in a broad range of circumstances.

Previously, we and others have examined the ability of bisbenzimide compounds to inhibit the replication of DNA viruses. Bisbenzimides bind adenine-thymine (A-T) rich sites in the minor groove of dsDNA [1,3]. The most well-known bisbenzimide compounds are the so-called Hoechst compounds that are used in diverse settings to identify DNA in cells using microscopy. Previously, it was demonstrated that Hoechst 33342 was a strong inhibitor of replication of the DNA virus vaccinia virus (VV) [4], the prototype poxvirus. This inspired our laboratory to examine further bisbenzimide compounds for antiviral activity, which led to the discovery of bisbenzimide compounds (RO-90-7501 and MRT00201423) that were strong inhibitors of the herpesvirus human cytomegalovirus (HCMV) [5], which has a DNA genome. Our further studies then demonstrated that Hoechst 33342, RO-90-7501 and MRT00210423 could strongly inhibit replication of both prototype (VV) and potential pandemic [monkeypox virus (MPXV)] poxviruses [6]. Activity of Hoechst 33342 against MPXV was later confirmed elsewhere [7]. It was also observed in our previous works that the binding of compounds to DNA appeared to be required for antiviral activity, as modification of compounds to prevent DNA binding led to a loss of either anti-HCMV or anti-poxvirus activity [5,6].

In all of the aforementioned studies, no obvious cellular cytotoxicity was observed with any bisbenzimide compound [4,6]. This was consistent with other studies from other laboratories that had examined cytotoxicity of bisbenzimide compounds and demonstrated no obvious toxicity using bisbenzimide compounds in either *in vitro* assays (RO-90-7501), experiments in murine models (Hoechst 33342) or human clinical trials (Hoechst 33258) [8,10].

We sought to progress the development of bisbenzimide compounds as antiviral compounds by identifying a bisbenzimide compound that had been used successfully in humans which could be repurposed as an antiviral compound. We selected Ridinilazole. Similar to other bisbenzimide compounds, Ridinilazole was first developed as an anti-cancer drug [11]. In contrast to many bisbenzimide compounds which possess an asymmetric structure, the structure of Ridinilazole was designed to be symmetric, in order to expand our understanding of what compounds had anti-cancer therapeutic activity while retaining the compounds’ ability to interact with DNA [11,12]. However, Ridinilazole had modest anti-proliferative activities (inhibitory concentration of 0.2–0.3 µM) against a panel of diverse human cancer cell lines (NCI-60 collection) and little anti-tumour activity in human cancer xenograft models [13]. Ridinilazole was subsequently repurposed and extensively tested in human clinical trials as an antibiotic targeting the bacterium *Clostridium difficile*. In the aforementioned clinical trials, it was found that oral administration of Ridinilazole had no obvious adverse effects to human patients and had highly efficacious antibiotic effects against *C. difficile* [14,16]. The United States Food and Drug Administration has previously granted Ridinilazole fast track status [11]. However, Ridinilazole is not yet widely used in clinical practice against *C. difficile* or any other bacteria, as Ridinilazole has not been shown to have greater antibiotic efficacy in clinical settings compared to current standards of care.

In this study, we set out to test the ability of Ridinilazole to inhibit replication of HCMV, including studies of how Ridinilazole potentially binds to HCMV genomes and examining how Ridinilazole acts to inhibit HCMV replication. Related issues were also considered, including the ability of Ridinilazole to inhibit the replication of other herpes viruses.

## Methods

### Cells

Human foreskin fibroblast (HFF) cells (clone Hs27) were obtained from American Type Culture Collection, no. CRL-1634 (ATCC, Manassas, VA). Vero cells were a gift from Donald Coen (Harvard). MDCK cells were a gift from Wendy Barclay (Imperial). All cells were maintained in complete media: Dulbecco’s modified Eagle’s medium (DMEM) (Gibco) containing 10% (v/v) FBS (Gibco), except for MDCK cells which were maintained in the aforementioned media supplemented with 0.1% penicillin/streptomycin solution.

### Human cytomegalovirus

HCMV strain Merlin (R1111) was generated from a bacmid containing deletions in ORFs encoding RL13 and UL128 to allow release of cell-free virus and was kindly provided by Richard Stanton (Cardiff) [17]. Strain AD169 was generously provided by Donald Coen (Harvard Medical School). Drug-resistant AD169 strains GDGrP53 [18] and PFArD100 [19] were obtained from the NIH AIDS reagents programme [donated by Donald Coen (Harvard Medical School)]. Virus stocks were produced in HFF cells. In all cases, titres were determined by serial dilution of viral supernatant onto HFF monolayers, which were then covered in DMEM containing 5% (v/v) FBS, antibiotics and 0.6% (w/v) methylcellulose. After incubation for 14 days, cells were fixed with 100% methanol and stained with crystal violet to count plaques in the infected cell monolayers. Titre was expressed as p.f.u. ml^−1^.

### Herpes simplex virus

Herpes simplex virus 1 (HSV-1) strain 17+ and HSV-2 strain 333 were gifts from Stacey Efstathiou (NIBSC) and David Knipe (Harvard Medical School), respectively. All virus stocks were generated by low multiplicity infections of Vero cells. Viral titre was determined as outlined in the preceding section by virus titration on Vero cells for 3 days.

### Influenza virus

Influenza A/Duck/Singapore/F119-3/97 (LPAI) was a gift from John McCauley (Francis Crick Institute). Stocks were obtained from low multiplicity infections of MDCK cells. Influenza infection was assessed using TCID50 assays, wherein Madin-Darby Canine Kidney (MDCK) cells were infected with virus in DMEM containing 0.1% penicillin/streptomycin solution, 0.1% BSA and 1 µg ml^−1^ L-1-tosylamido-2-phenylethyl chloromethyl ketone (TPCK)-treated trypsin. At 72 h post-infection, infected cells were fixed with 4% formaldehyde solution and stained with crystal violet.

### Compounds

RO-90-7501 (CAS number 293762-45-5) was purchased from Tocris. MRT00210423 was synthesized by LifeArc (formerly MRC Technology) as previously described [5]. Ridinilazole (previously known as SMT19969) (CAS number 308362-25-6) was purchased from Cambridge Bioscience (lot number 31791). Ganciclovir (CAS number 82410-32-0) was purchased from Insight Biotechnology. All compounds were resuspended in DMSO.

### Virus yield assays

HFF cells were plated at 5×10^4^ cells per well in 24-well plates. After overnight incubation to allow cell attachment, cells were either infected with 5×10^4^ p.f.u. of HCMV (m.o.i.=1). HCMV viruses used in each experiment are indicated in the text and figure legends. In each case, after virus adsorption for 1 h at 37 °C, cells were washed and incubated with 0.5 ml of media in the presence or absence of drug throughout virus replication. Infected cells were incubated for 96 h at 37 °C before supernatant was removed from cells for analysis of virus titre by plaque counting, as described above.

### MTT assays for cell viability

HFF were seeded at high (5×10^3^ cells per well) or low (5×10^2^ cells per well) numbers of cells per well into 96-well plates. High numbers of cells (5×10^3^ cells per well) were to assess cell viability, whereas low numbers of cells (5×10^2^ cells per well) were to assess both cell viability and cell proliferation. After overnight incubation to allow cell attachment, cells were treated for 96 h with DMSO or compounds as indicated in the figures and text. 3-(4,5-dimethylthiazol-2-yl)-2,5-diphenyltetrazolium bromide (MTT) assays were carried out on cells in the wells of 96-well plates according to the manufacturer’s instructions (GE Healthcare). The ability of cellular NAD(P)H-dependent cellular oxidoreductase enzymes to reduce the tetrazolium dye MTT to formazin was measured in a colorimetric assay, read on a FLUOstar Omega Microplate Reader.

### Cell counting assays

To count cells, HFF cells were seeded at high or low concentrations of cells per well into 24-well plates. High numbers of cells (5×10^4^ cells per well) were to assess cell viability, whereas low numbers of cells (5×10^3^ cells per well) were to assess both cell viability and cell proliferation. After overnight incubation to allow cell attachment, cells were treated for 96 h with 10 µM Ridinilazole or the same volume of DMSO. At 96 h post-treatment, cells were removed from wells using trypsin, re-suspended in complete cell media and counted in the presence of trypan blue using a Countess Automated Cell Counter (Invitrogen) using the manufacturer’s instructions.

### Bioinformatic analysis of putative bisbenzimide binding sites in viral genomes

This analysis was carried out as previously described [5]. Briefly, genome sequences in fasta format and gene features in gff format were downloaded from GenBank with the help of NCBI’s eutils API. Using a custom FIND_MOTIF programme, nucleotide motifs were extracted from genome sequence files and their corresponding gene features from gff file. The locations of the motifs were then compared with genome features table file using an AWK script to find whether the motifs were part of any coding region. Final results were stored in a specified output file (Microsoft Excel). The complete analysis pipeline along with the source code is available on the GitHub page (https://github.com/vbsreenu/find-motif). GenBank accession numbers used in this study: AD169-BK000394.5, Merlin–NC_006273.2, Strain 17–GCF_000859985.2 and Strain 333–KP192856.1.

### Prediction of compound lipophilicity

The non-ionic consensus partitioning coefficient (LogP) of compounds listed in the text was determined using the AxonChem Marvin cheminformatics suite. In all cases, default conditions were used; calculations assumed Cl− and Na+ K+ concentrations of 0.1 mol dm^−3^ each. Tautomerization or resonance was not considered.

### Western blotting

Conditions under which HFF cells were infected and treated with compounds or DMSO are detailed in the text and figure legends. Cell lysate was prepared for western blotting by washing the cells once in PBS (SIGMA), suspending the cells directly in 2× Laemmli buffer containing 5% *β*-mercaptoethanol and incubating at 95 °C for 5 min.

Proteins were separated on 8 or 10% (v/v) polyacrylamide gels and transferred to a Hybond-ECL membrane (Amersham Biosciences) using a semi-dry protein transfer apparatus. The membranes were blocked at room temperature for at least 90 min using TBS containing 0.1% Tween-20 and 5% dried powdered milk (TBSTM) and then incubated overnight at 4 °C in TBSTM plus primary antibodies: antibodies recognizing HCMV IE1/2 (antibody CH160), UL57 (antibody CH167), UL44 (antibody 10D8) or pp28 (antibody CH19), (all Virusys, 1:1,000 dilution) or *β*-actin (SIGMA, 1:5,000 dilution).

After incubation in TBSTM with primary antibodies, the membrane was washed extensively with TBST and incubated for 60 min at room temperature with TBSTM containing Dylight 800 Goat Anti-Mouse IgG (Abbkine, A23910) to detect primary antibodies. After further washing with TBST and TBS, antibodies were detected using an Odyssey Cx machine.

### Electron microscopy preparation and imaging of infected cells

To prepare cells for imaging, HFF cells (1×10^5^ or 2×10^5^ cells) were infected with m.o.i. 1 of Merlin (R1111) for 96 h in 24-well plates, then detached from plates using trypsin and washed twice with PBS (SIGMA). Cells were then washed once in Sorensen’s phosphate buffer (SPB), resuspended in 4% glutaraldehyde in SPB and incubated overnight at 37 °C. Fixative was then removed, and cells were washed three times in SPB before being resuspended in SPB and prepared for imaging. Cells were prepared for imaging and imaged at Queen Mary’s, University of London, Transmission Electron Microscopy Facility under a commercial agreement. To image cells, cells were fixed in 4% buffered glutaraldehyde and embedded in 2% low melting point agarose. Agarose was cut into 1 mm^3^ blocks and embedded in Araldite resin (Agar Scientific Ltd., Essex, UK). Resin blocks were ultrathin sectioned using a Reichert-Jung Ultracut E Ultramicrotome (Leica) and sections were examined by transmission electron microscopy (TEM) using a JEM1400F (JEOL, UK) microscope at 120 kV. TEM images were captured using a high-sensitivity sCMOS Matataki Flash camera. Cell maps encompassing the entire nucleus and cytoplasm of nine cells in each condition selected at random were created at 20k magnification using the ultra-wide area montage system Limitless Panorama (JEOL).

## Results

### Inhibition of HCMV replication by Ridinilazole

**Fig. 1.**
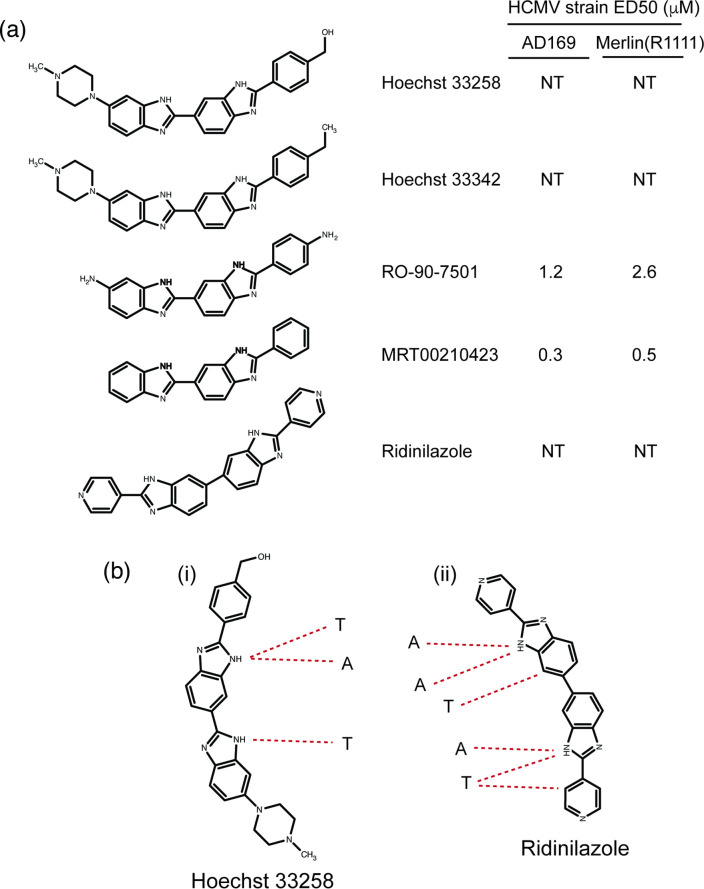
Structure, antiviral activity and A-T pair binding of bisbenzimide compounds. (**a**) Structures of bisbenzimide compounds are shown on the left of the figure. These include asymmetric bisbenzimides such as the commonly used Hoechst compounds 33258 and 33342 and previously studied compounds RO-90-7501 and MRT00201423. Also shown is the structure of the symmetric bisbenzimide compound Ridinilazole. On the right are listed the ED_50_ of compounds against HCMV strains AD169 and Merlin (R1111) [5]. NT: Not tested in our previous studies [5]. (**b**) Hydrogen bonding (red dotted lines) between amine groups in (**i**) Hoechst 33258 and (ii) Ridinilazole to adenine (A) and thymine (T) nucleotides in the minor groove of DNA, as demonstrated in previous studies [1,12].

The structures of asymmetric and symmetric bisbenzimide compounds are shown in Fig. 1(a). All have the common feature of binding to A-T base pairs in the minor groove of DNA via amine groups in each compound (Fig. 1b). We previously demonstrated that bisbenzimide compounds RO-90-7501 and MRT002010423 were effective inhibitors of different HCMV strains [5]. The ED_50_ of both compounds against the high-passage HCMV strain AD169 and the low-passage HCMV strain Merlin (R1111), all between 0.1 and 2.6 µM [5], are shown in Fig. 1(a).

**Fig. 2.**
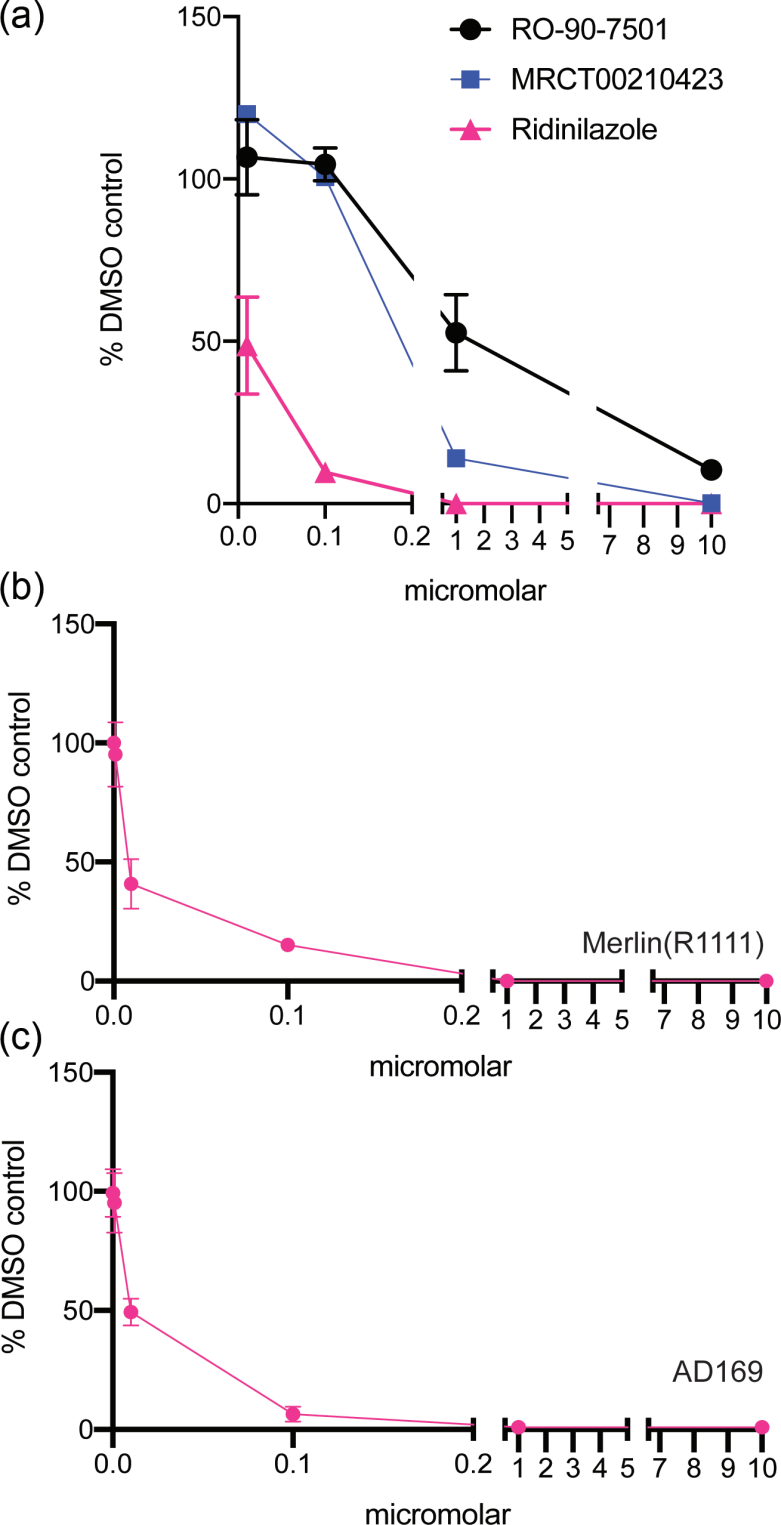
Anti-HCMV effects of bisbenzimide compounds. (**a**) In virus yield assays, HFF cells were infected with HCMV strain Merlin (R1111) at m.o.i. 1 and treated with concentrations of bisbenzimide between 10 and 0.01 µM or the corresponding volume of DMSO. Virus production at 96 h post-infection is shown as the percentage of infectious virus in the presence of bisbenzimide compared to the appropriate DMSO control. (**b) and (c**) In virus yield assays, HFF cells were infected with either HCMV strain (**b**) Merlin (R1111) or (**c**) AD169 and treated with concentrations of bisbenzimide between 10 and 0.0001 µM or the corresponding volume of DMSO. Virus production at 96 h post-infection is shown as the percentage of infectious virus in the presence of bisbenzimide compared to the appropriate DMSO control. In each figure, the data points and error bars in each panel represent the mean of three independent experiments and the sd of those experiments, respectively. At some data points, the error bars are too small to be represented on the figure.

Using virus yield assays, we compared the ability of Ridinilazole (structure shown in Fig. 1) to inhibit replication of Merlin (R1111) compared to RO-90-7501 and MRT00210423 (Fig. 2a). The ED50 values of both RO-90-7501 and MRT00210423 against Merlin (R1111) were 1.8 and 0.18 µM, respectively, which were similar to ED50 values previously reported for those compounds (Figs 1 and 2a). However, we observed that the ED50 value of Ridinilazole was considerably below either RO-90-7501 or MRT00210423 at ~0.01 µM, indicating that this bisbenzimide compound was a more effective inhibitor of HCMV replication compared to those previously tested.

To more accurately understand the EC50 value of Ridinilazole against HCMV, we performed virus yield assays of a greater range of compound concentrations using either Merlin (R1111) or AD169 (Fig. 2b, c). Similar to the data shown in Fig. 2(a), the EC50 values of Ridinilazole against both HCMV strains were ~0.01 µM.

### Investigation of cell viability and cell division in the presence of Ridinilazole

**Fig. 3.**
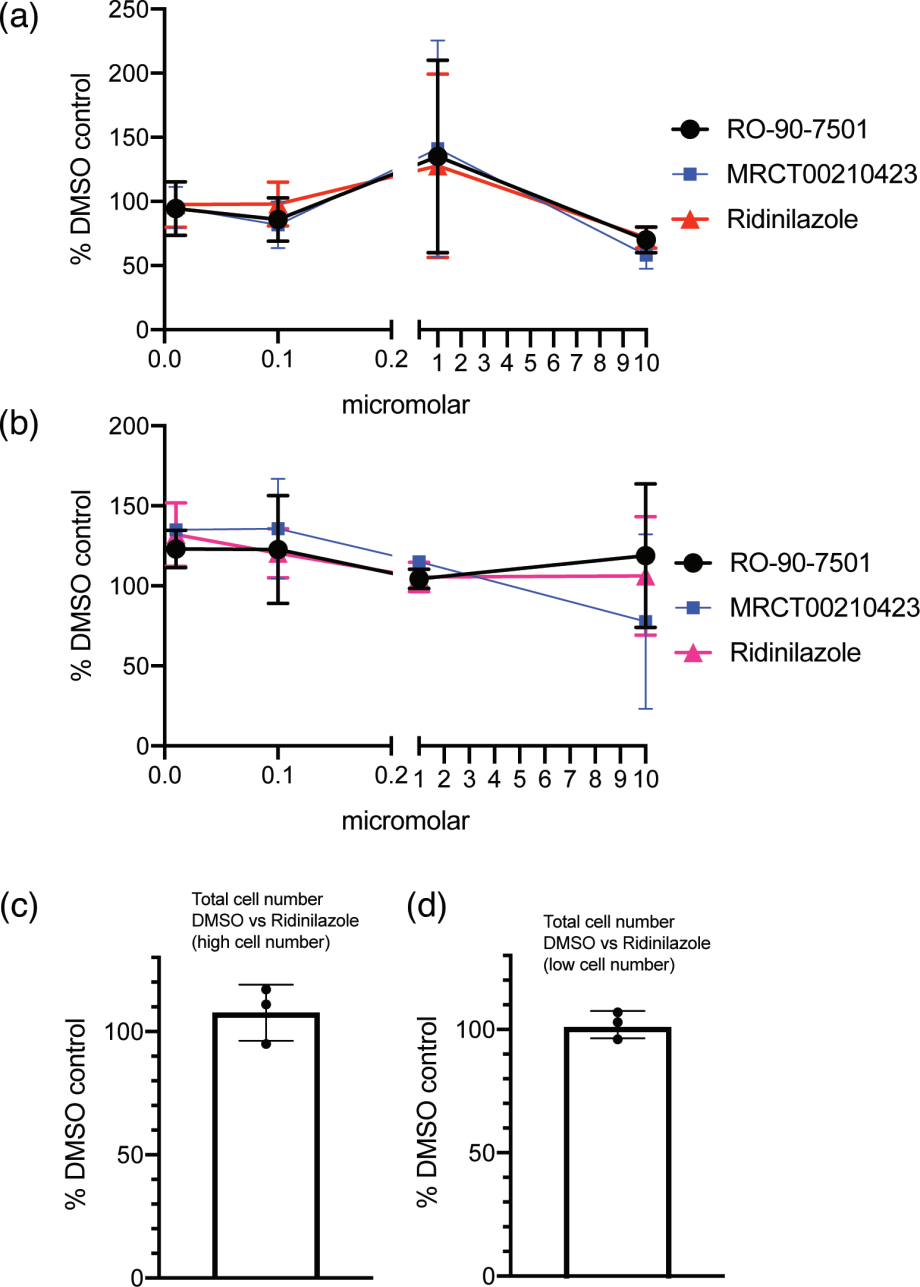
Cell viability and cell division in the presence of bisbenzimide compounds. (**a) and (b**) A high or low concentration of HFF cells (A and B, respectively; see Methods) was treated for 96 h with concentrations of bisbenzimide compounds between 10 and 0.01 µM or the corresponding volume of DMSO and then examined using an MTT assay. In each figure, the data points and error bars in each panel represent the mean of three independent experiments and the sd of those experiments, respectively. At some data points, the error bars are too small to be represented on the figure. (**c) and (d**) Uninfected HFF cells plated at (**c**) high or (**d**) low numbers were treated with 10 µM Ridinilazole or the same volume of DMSO for 96 h. Cell number in each condition was then investigated using an automated cell counter. The percentage of Ridinilazole-treated cells compared to DMSO-treated cells was calculated. In each figure, data are representative of three independent experiments (black data points) and presented as the average (block) and sd (error bars) of the data.

To exclude the possibility that the decrease in HCMV yield in the presence of Ridinilazole seen in Fig. 1 was due to loss of cell viability, we performed cellular viability assays (MTT assays) on uninfected cells compared to cells treated with DMSO. In these assays, high concentrations of uninfected cells were tested to examine cell viability (Fig. 3a), and low concentrations of cells were tested to examine both cell viability and cell division (Fig. 3b). We found no obvious concentration-dependent effect on uninfected cell viability or cell division for any compound tested in MTT assays, including at concentrations that had obvious antiviral effects. Similarly, when we directly counted high and low numbers of uninfected cells in the presence of either DMSO or 10 µM Ridinilazole (Fig. 3c, d, respectively), we found that the presence of Ridinilazole did not have any obvious effect on cell numbers in any experiment.

### Reversible inhibition of HCMV replication by Ridinilazole

**Fig. 4.**
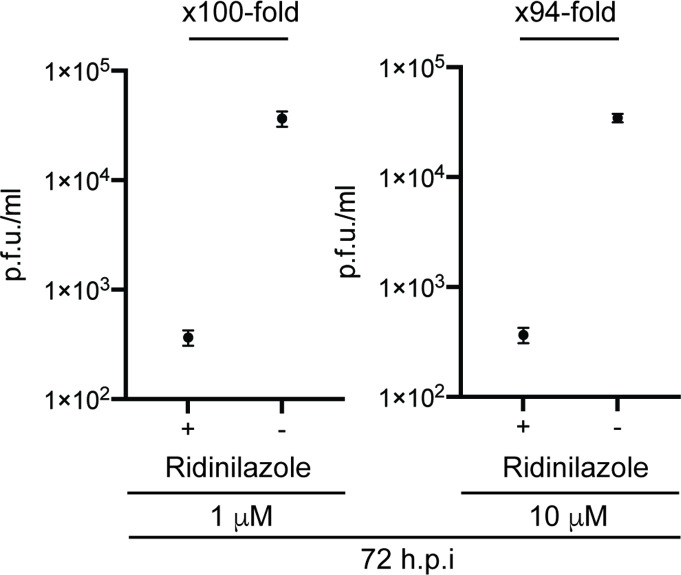
Reversible inhibition of HCMV by Ridinilazole. HFF cells were infected with HCMV strain Merlin (R1111) at m.o.i. 1 and treated with either 1 or 10 µM Ridinilazole. After 72 h post-infection, supernatant containing compound was removed and replaced with cell culture media containing the same concentration of compound (+) or cell culture media lacking compound (−). Infected cells were then incubated for a further 96 h. Viral titre from infections is shown as p.f.u. ml^−1^. The data are representative of three independent experiments and presented as the mean (black square) and sd (error bars) of the data. The fold difference in the mean values is shown above each figure.

It was unknown if bisbenzimide inhibition of HCMV was reversible. Therefore, we tested if inhibition of HCMV replication by Ridinilazole was reversible. We examined this possibility by infecting cells in the presence of Ridinilazole and then continuing incubation of infected cells in the presence (Ridinilazole +, Fig. 4) or absence of Ridinilazole (Ridinilazole −, Fig. 4). Removal of Ridinilazole from infected cells treated with Ridinilazole resulted in an increase in HCMV production compared to those infected cells maintained in the presence of the compound. Therefore, the antiviral effect of Ridinilazole appeared to be reversible when the compound was removed from HCMV-infected cells.

### Antiviral effects of Ridinilazole compared to Ganciclovir

**Fig. 5.**
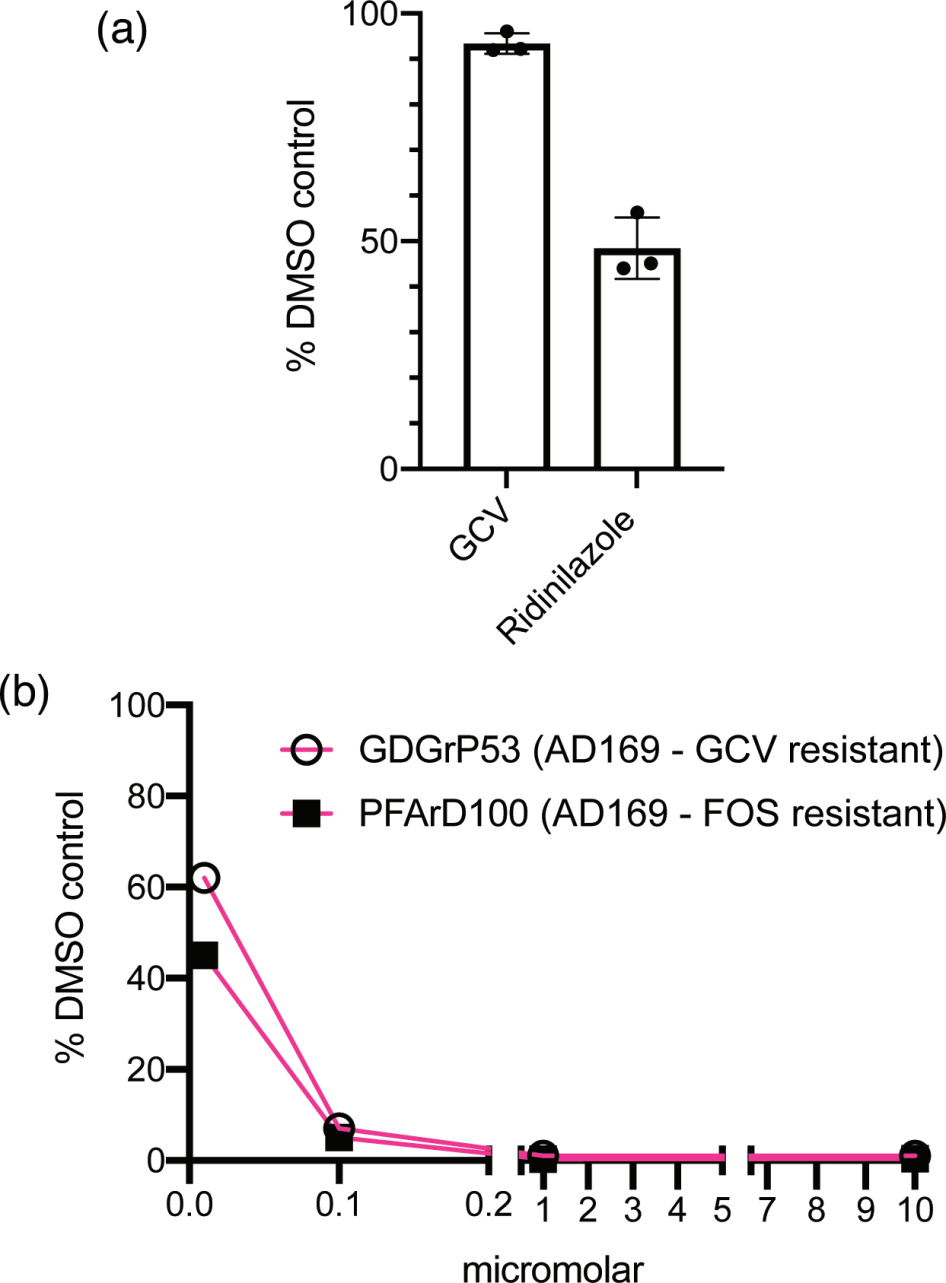
Antiviral effects of Ganciclovir and antiviral effects of Ridinilazole on HCMV drug-resistant strains. (**a**) In virus yield assays, HFF cells were infected with HCMV strain Merlin (R1111) at m.o.i. 1 and treated with either 0.01 µM Ganciclovir or Ridinilazole or the corresponding volume of DMSO. (**b**) In virus yield assays, HFF cells were infected with HCMV strains GDGrP53 (Ganciclovir resistant) or PFArD100 (Foscarnet resistant) and treated with concentrations of Ridinilazole between 10 and 0.01 µM or the corresponding volume of DMSO. In both figures, virus production at 96 h post-infection is shown as the percentage of infectious virus in the presence of bisbenzimide compared to the appropriate DMSO control. In figure (**a**), the data points and error bars represent the mean of three independent experiments and the sd of those experiments, respectively. (GCV – ganciclovir, FOS – Foscarnet).

We noted that the ED50 of Ridinilazole in our experiments (0.01 µM) was notably lower than the reported ED50 of the most commonly used anti-HCMV drug, Ganciclovir (~0.1–10 µM) [20,21]. Therefore, Ridinilazole may have had greater antiviral activity than ganciclovir. To directly test this proposal, we compared the antiviral effects of Ridinilazole to ganciclovir at a low concentration of both compounds (0.01 µM) (Fig. 5a). Consistent with previous data, Ganciclovir had little antiviral effect at low concentration, whereas the antiviral effect of Ridinilazole was similar to that seen in the experiments above (Fig. 2). Thus, in our assays, Ridinilazole had greater antiviral effects than the most commonly used anti-HCMV drug.

### Action of Ridinilazole on drug-resistant HCMV strains

We also tested the ability of Ridinilazole to inhibit replication of AD169 strains of HCMV containing mutations causing single amino acid changes in the HCMV DNA polymerase catalytic subunit that confer resistance to either Ganciclovir (virus GDGrP53) [18] or Foscarnet (virus PFArD100) [19]. We observed that both drug-resistant AD169 strains were inhibited by Ridinilazole to the same extent as wild-type AD169, with ED50 values of ~0.01 µM (compare data in Figs 2c and 5b). Therefore, Ridinilazole action on HCMV was not obviously affected by drug resistance mutations in the HCMV genome, and Ridinilazole could be useful in treating HCMV drug-resistant infections.

### Investigation of Ridinilazole activity against RNA and DNA viruses

To better understand how Ridinilazole could be more widely applied as an antiviral compound, we then tested the antiviral activity of Ridinilazole against several viruses, including those viruses with either RNA (influenza) or DNA genomes (herpesviruses).

**Fig. 6.**
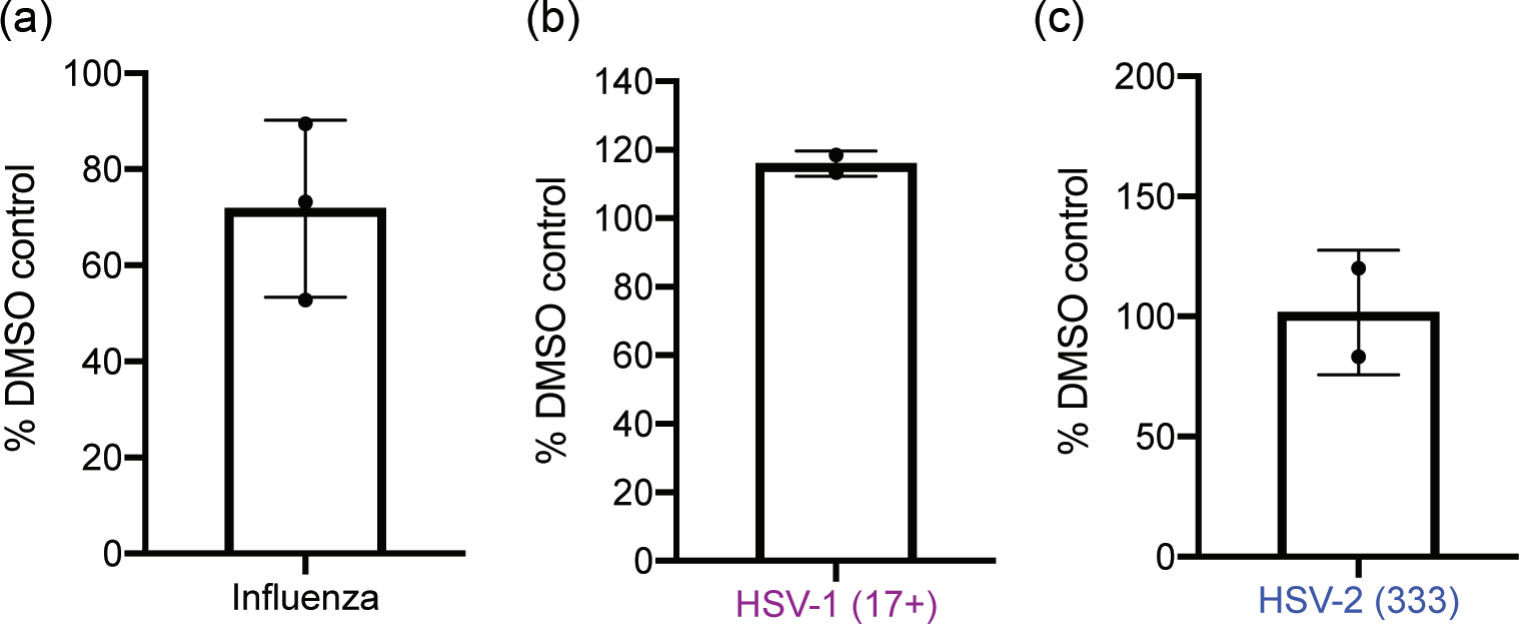
HSV and influenza replication in the presence of Ridinilazole. (**a**) MDCK cells were infected with influenza A at an m.o.i. of 0.01 in the presence of 10 µM Ridinilazole or the equivalent volume of DMSO. Viruses were harvested at 72 h post-infection, and viral titre (TCID50) was determined by titration of viral supernatant on MDCK cells. Data are presented as the percentage of infectious virus in the presence of Ridinilazole compared to the appropriate DMSO control. The data from three independent experiments were presented in each figure. The bar and error bars represent the mean and sd of the data, respectively. (**b) and (c**) HFF cells were infected with (**a**) HSV-1 strain 17+ or (**b**) HSV-2 strain 333 at an m.o.i. 1 in the presence of 10 µM Ridinilazole or the equivalent volume of DMSO. Viruses were harvested at 48 h post-infection, and viral titre (p.f.u. ml^−1^) was determined by titration of viral supernatant on Vero cells. Data are presented as the percentage of infectious virus in the presence of Ridinilazole compared to the appropriate DMSO control. The data from two independent experiments were presented in each figure. The bar and error bars represent the mean and range of the data, respectively.

When influenza-infected cells were treated with either DMSO or a high concentration of Ridinilazole (10 µM), we observed very poor inhibition of virus replication in the presence of Ridinilazole (Fig. 6a). Thus, Ridinilazole was unlikely to be a broad-spectrum antiviral agent, able to inhibit replication of both DNA and RNA viruses.

We then tested the possible antiviral effects of Ridinilazole against the herpesviruses HSV-1 (strain 17+) and HSV-2 (strain 333). Upon infection in the presence of a high concentration of Ridinilazole (10 µM), we found no obvious difference in the ability of either HSV-1 or HSV-2 to replicate in the presence of Ridinilazole compared to control infections performed in the presence of DMSO (Fig. 6b, c). Thus, Ridinilazole was unlikely to be a broad-spectrum antiviral agent, able to inhibit replication of all DNA viruses.

### Analysis of putative bisbenzimide binding sites in HCMV genomes

We next investigated compound targets by examining the relationship between antiviral activity of bisbenzimide compounds and the number of putative bisbenzimide binding sites in HCMV genomes. Previous reports of Hoechst 33258 DNA footprinting assays have identified DNA binding sites [3] that are likely to be shared by asymmetric bisbenzimides [1,2], such as RO-90-7501 and MRT00210423. A recent report using DNA footprinting has directly identified DNA binding sites for Ridinilazole on DNA [12]. Typical of bisbenzimide compounds, A-T base pairs were prominent in each compound binding motif identified in the aforementioned studies. The report of Hoechst 33258 binding to DNA identified with DNA motifs AAATT, ATTTT, AATAA, TATA, TTAATG, GTTTAT and TTTCT, with a modest preference for binding in that order [3]. It was reported that Ridinilazole binds to the DNA motifs AATTT, ATTTT, AATT, TAATT, ATATT, TTTTT and AAATTTA with a modest preference for AATTT and ATTTT [12]. The only motif recognized by both asymmetric and symmetric bisbenzimides appeared to be ATTTT.

**Fig. 7.**
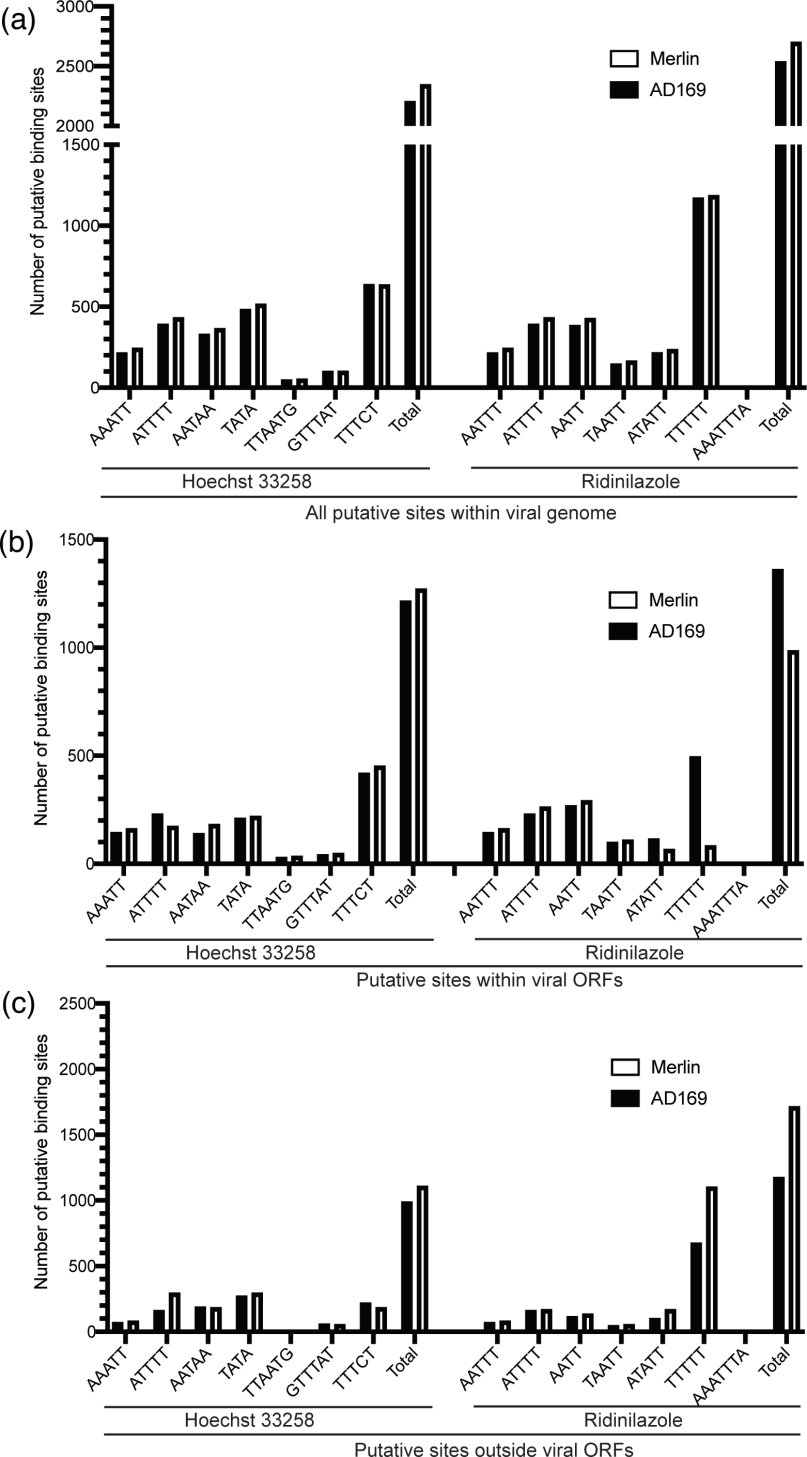
Bioinformatic analysis of putative compound binding sites in HCMV genomes. A bioinformatics approach (outlined in the Methods) was used to count the number of putative binding sites of asymmetric bisbenzimides or Ridinilazole in HCMV genomes AD169 or Merlin. (**a**) Total number of sites within genomes. (**b**) Number of sites within viral ORFs. (**c**) Number of sites outside viral ORFs.

Using our previously described bioinformatics approach to find DNA motifs within DNA viral genomes [5], we identified the number of either putative RO-90-7501 and MRT00210423 binding sites or putative Ridinilazole binding sites within Merlin and AD169 genomes. Overall, we found that the total number of putative binding sites for RO-90-7501 or MRT00210423 was similar to those for Ridinilazole in HCMV genomes (Fig. 7a). To extend this analysis, we also examined the number of putative binding sites within HCMV ORFs and outside of HCMV ORFs (Fig. 7b, c, respectively). There was no obvious difference between the compounds in this analysis. Therefore, the number or position of putative bisbenzimide binding sites in the HCMV genome was not obviously related to antiviral activity.

There were modest differences in the number of Ridinilazole binding sites within the Merlin and AD169 genomes, likely due to the greater length of the Merlin genome compared to the AD169 genome (235,646 and 230,290 bp, respectively). However, this did not obviously affect the antiviral activity of Ridinilazole against these HCMV strains (Fig. 2).

### Analysis of compound lipophilicity

If differences in bisbenzimide compound antiviral activity were not due to interaction with DNA, we examined if antiviral activity was related to lipophilicity. Previous investigation of Hoechst compound inhibition of poxvirus replication had noted that antiviral activity of different Hoechst compounds against VV correlated with increased lipophilicity of compounds [4], suggesting that solubility of bisbenzimide compounds was a factor in their antiviral activity. We assessed the lipophilic properties of our bisbenzimide compounds and found that RO-90-7501, MRT00210423 and Ridinilazole had LogP values of 2.94, 4.60 and 3.81, respectively. The lipophilicity of Ridinilazole was not greater than that of MRT00210423, suggesting that greater antiviral effect of Ridinilazole compared to other bisbenzimide compounds was not due to greater solubility of this compound compared to the others tested.

### Analysis of putative Ridinilazole binding sites in HCMV and HCMV genomes

**Fig. 8.**
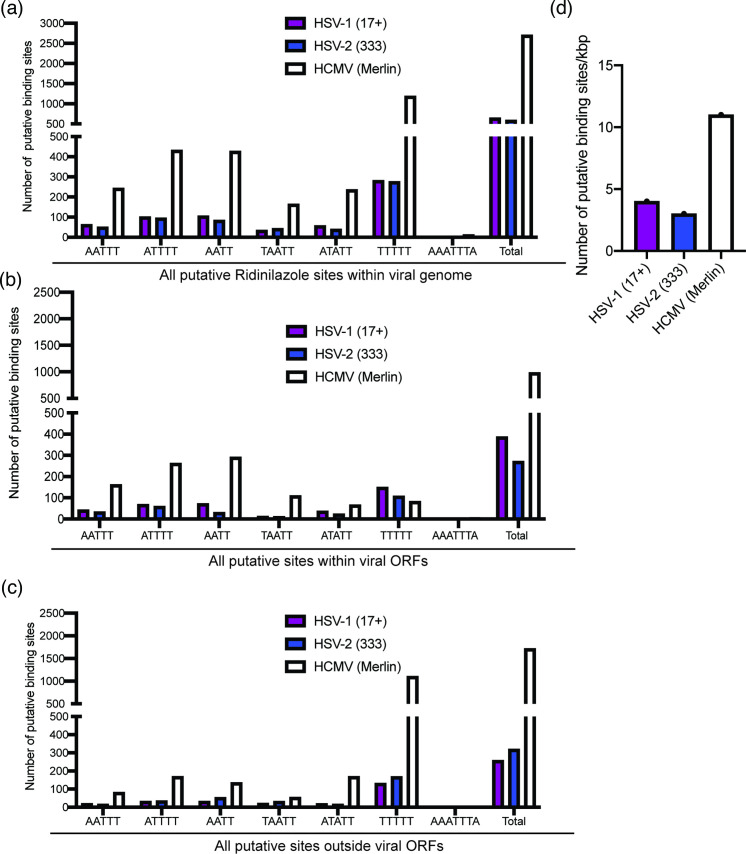
Bioinformatic analysis of putative compound binding sites in HSV and HCMV genomes. A bioinformatics approach (outlined in the Methods) was used to count the number of putative binding sites of Ridinilazole in HSV genomes 17+ or 333. (**a**) Total number of sites within genomes. (**b**) Number of sites within viral ORFs. (**c**) Number of sites outside viral ORFs. (**d**) Number of sites per kbp of genome.

To better understand Ridinilazole interaction with viral genomes, we compared the putative Ridinilazole binding sites in HCMV genomes compared to those in HSV genomes. Similar numbers of putative Ridinilazole binding motifs were found in both 17+ and 333 genomes. However, the overall number of motifs in both HSV genomes was considerably fewer than in either HCMV strain (Fig. 8a–d). This was likely due to the differences in length between HCMV and HSV genomes (HSV-1 strain 17+: 152,222 bp; HSV-2 strain 333: 153,333 bp; HCMV strain Merlin: 235,646 bp). Therefore, it is possible that a factor in the ability of Ridinilazole to inhibit HCMV replication, but not HSV replication, was the greater opportunity for Ridinilazole to bind to HCMV genomes compared to HSV genomes.

### HCMV protein expression in the presence of Ridinilazole

**Fig. 9.**
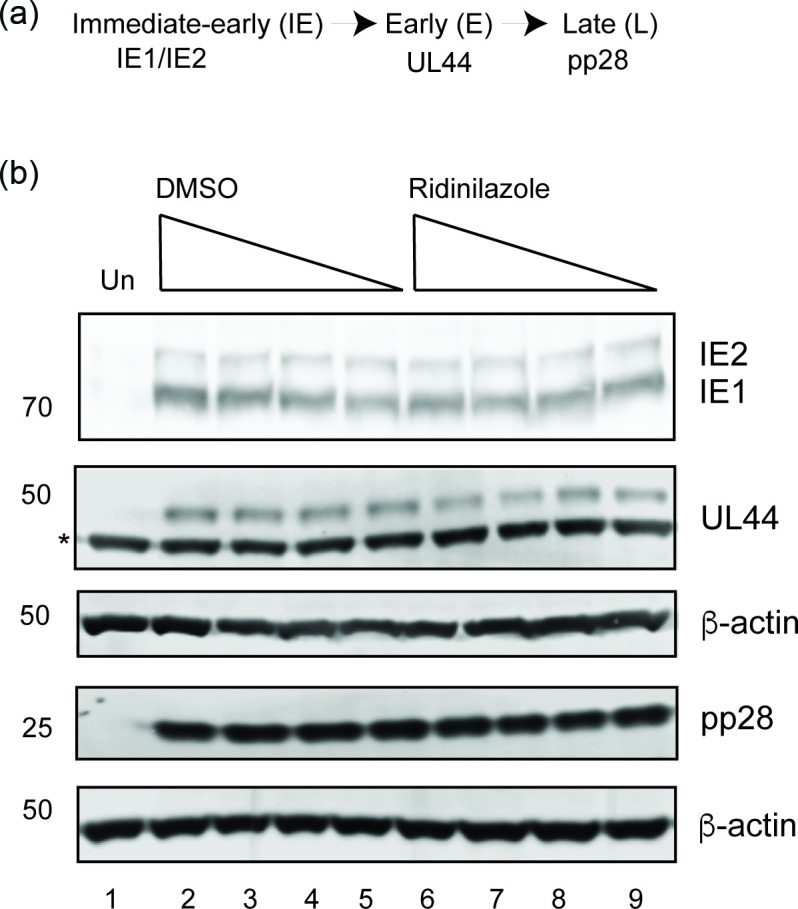
Western blotting of infected cell lysate. (**a**) Scheme of protein expression in HCMV-infected cells. (**b**) HFF cells were infected with HCMV strain Merlin (R1111) at m.o.i. 1 and treated with concentrations of Ridinilazole between 10 and 0.01 µM (10-fold dilution series) or the corresponding volume of DMSO, as indicated in the figure. At 96 h post-infection, cell lysate was prepared for western blotting. Uninfected cell lysate was also prepared for western blotting at 96 h post-infection. A background band detected by an antibody recognizing UL44 is marked with a star. Proteins recognized by the antibodies used in the experiment are indicated to the right of the figure, and the positions of molecular weight markers (kDa) are indicated to the left of the figure.

Next, we investigated how Ridinilazole inhibited HCMV replication by assaying HCMV protein expression in the presence or absence of the compound using western blotting. Like other herpesviruses, HCMV protein expression is dictated by a transcriptional cascade, dependent on viral DNA synthesis, that produces immediate-early (IE), early (E) and late (L) proteins. We used antibodies recognizing representatives of IE, E and L protein expression (IE1/IE2, UL44 and pp28, respectively) (Fig. 9a). We found that the presence of Ridinilazole had no obvious effect on the expression of any HCMV protein (Fig. 9b). Therefore, Ridinilazole did not obviously restrict the HCMV protein expression in infected cells. Also, as Ridinilazole had no obvious effect on the presence of pp28, whose expression is dependent on viral DNA synthesis [22], this data indicated that the presence of Ridinilazole did not obviously compromise HCMV DNA synthesis, similar to our previous observations that either RO-90-7501 or MRT00210423 had very little or no effect on HCMV DNA synthesis [5].

### Electron microscopy analysis of HCMV-infected cells treated with Ridinilazole

**Fig. 10.**
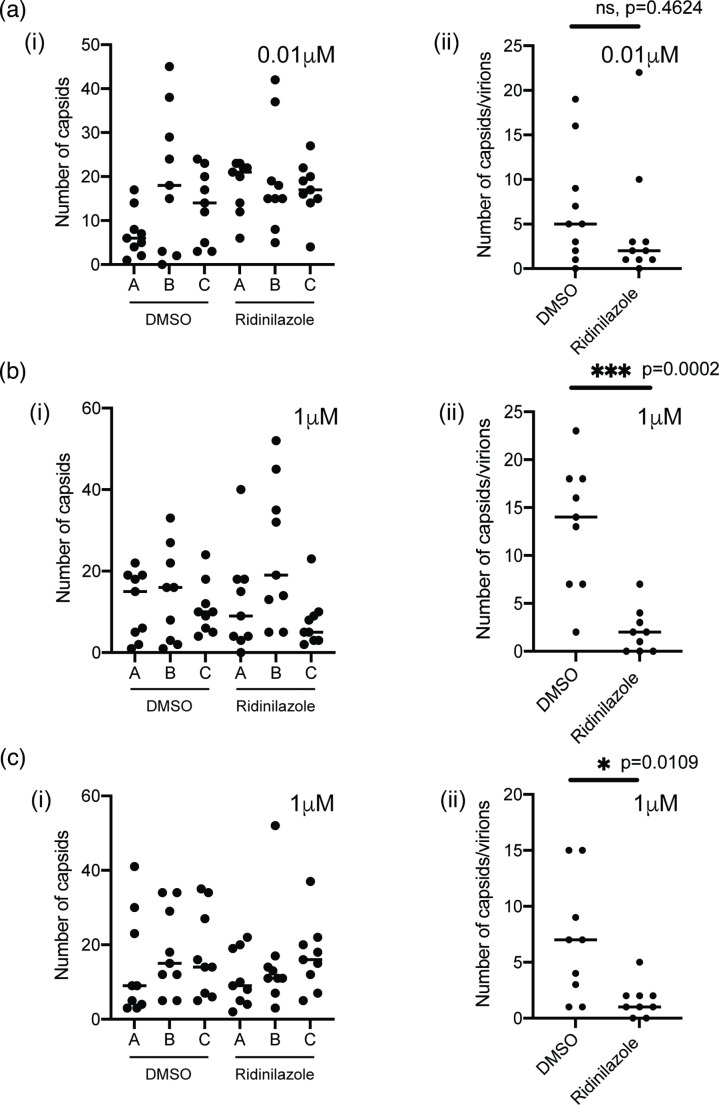
Electron microscopy of HCMV-infected cells. HFF cells were infected with HCMV strain Merlin (R1111) at m.o.i. 1 and treated with concentrations of Ridinilazole of (**a**) 0.01 µM or (**b**) and (**c**) 1 µM or the corresponding volume of DMSO. At 96 h post-infection, cells were prepared for analysis using electron microscopy. In each independent experiment (**a–c**), nine cells were selected at random, and images were taken to capture the number of capsids in (**i**) the entire nuclear or (ii) the entire cytoplasmic areas of each cell. Statistical relevance was examined using an unpaired, two-tailed, Student's t-test. In (**a**), no statistically relevant difference was found between any capsid type in the presence or absence of Ridinilazole in A(i), B(i) or C(i) (data not shown). In (**b**), statistically relevant differences reflected *P*≤0.05. (ns=not statistically different).

Our previous investigation of HCMV replication in the presence of either RO-90-7501 or MRT002010423 revealed that these compounds, like Ridinilazole, did not compromise HCMV protein expression [5]. However, analysis of HCMV capsid production in the presence of either RO-90-7501 or MRT002010423 using TEM pointed to a lack of HCMV capsid production in the nucleus in the presence of these compounds [5]. Therefore, to further understand how Ridinilazole inhibited HCMV replication, we assayed HCMV capsid production in the presence of either DMSO or Ridinilazole using electron microscopy (Fig. 10). Initially, we assayed production of A capsids (non-productive forms thought to result from failed packaging of viral genomes), B capsids (productive intermediates that contain a scaffolding protein) and C capsids (assembled forms in which the scaffolding protein has been removed and replaced with viral DNA) [23] in the nucleus using the ED50 value we determined for Ridinilazole (0.01 µM) (Fig. 2). Cells were selected for analysis at random, and images of cells were gathered to visualize the entire area of both the nucleus and the cytoplasm of each cell.

We found no statistically relevant difference between the number of any capsid type produced in the presence and absence of Ridinilazole [Fig. 10a(i)]. However, we also assayed the number of capsids in the cytoplasm. Here, the data suggested that the presence of Ridinilazole decreased the number of cytoplasmic capsids, but was not statistically relevant [Fig. 10b(ii)]. To confirm these observations, experiments were conducted with a high concentration of Ridinilazole (1 µM), which inhibited nearly all HCMV replication in our assays (Fig. 2). Again, the presence of Ridinilazole had no statistically relevant effect on production of any capsid type in the nucleus [Fig. 10b(i), c(i)], but it did notably decrease the number of cytoplasmic capsids [Fig. 10b(ii)]. Therefore, inhibition of HCMV replication by Ridinilazole was not due to loss of protein production (Fig. 9) or, like RO-90-7501 and MRT002010423, loss of nuclear capsid production [5]. Rather, loss of HCMV replication in the presence of Ridinilazole was likely due to loss of cytoplasmic capsids (Fig. 10).

Additionally, the presence of Ridinilazole had no obvious effect on the production of DNA-containing capsids [Fig. 10a(i), b(ii) and c(ii)]. This further emphasized that, like RO-90-7501 or MRT00210423 [5], Ridinilazole was not obviously inhibiting HCMV DNA synthesis in our experiments.

## Discussion

HCMV remains a major factor in congenital disease and transplant rejection in humans [24,25], and currently used anti-HCMV drugs have many shortcomings, including toxicity and drug resistance [24,26, 27]. Here, we find that the symmetric bisbenzimide Ridinilazole was a considerably more efficacious inhibitor of HCMV replication compared to the asymmetric bisbenzimide compounds RO-90-7501 and MRT00210423. We found that the EC50 value of Ridinilazole (0.01 µM) was considerably lower than the ED50 values reported for the most commonly used anti-HCMV drug, Ganciclovir (~0.1–10 µM) [20,21]. Moreover, Ridinilazole was an efficacious inhibitor of HCMV viruses resistant to either Ganciclovir or another frontline anti-HCMV drug, Foscarnet.

It remains unknown why Ridinilazole was a more efficacious inhibitor of HCMV compared to the other bisbenzimide compounds that we have previously tested. We found that there was no obvious correlation between anti-HCMV effects, lipophilicity of the compounds studied or the number of putative RO-90-7501, MRT00210423 and Ridinilazole binding sites in HCMV genomes. However, we cannot exclude the possibility that there are as yet undiscovered DNA motifs that Ridinilazole, but not RO-90-7501 or MRT00210423, can bind to. Alternatively, there may be as yet unrecognized differences in the affinity for compounds to bind DNA.

Importantly, the number of bisbenzimide binding sites in viral DNA genomes may not be as important as the ability of compounds to access those sites. We have previously speculated that the different antiviral effects of RO-90-7501 and MRT00210423 are due to the differences in the ability of the compounds to enter the minor groove of DNA [5]. Detailed structural analysis of Ridinilazole binding to the minor groove of DNA indicated no obvious impediment in the structure of Ridinilazole to access to the minor groove of DNA [12]. Therefore, the anti-HCMV effects of Ridinilazole could be the result of both a large number of binding sites within HCMV genomes and the ability of the compound to access the minor groove of DNA.

It remains unclear what mechanisms of action facilitate the antiviral activity of Ridinilazole against HCMV. It is unlikely that bisbenzimide compounds directly inhibit HCMV DNA replication. We have previously demonstrated that RO-90-7501 and MRT00210423 had little or no direct effect on HCMV synthesis [5]. Here, we find that Ridinilazole had no effect on the production of a viral protein (pp28), whose expression requires DNA synthesis [22], and had no effect on the production of DNA-containing capsids in the nucleus. Previously, we found that RO-90-7501 had no effect on HCMV protein production, but did inhibit production of nascent HCMV capsids in the infected cell nucleus [5]. In contrast, Ridinilazole had no obvious effect on HCMV protein production or production of nascent HCMV capsids in the nucleus, but decreased the number of HCMV capsids undergoing maturation to virions in the cytoplasm. We think it possible that the interaction of bisbenzimide compounds with HCMV DNA results in multiple inhibitory effects that lead to the inhibition of capsid or virion production. It remains possible that binding of Ridinilazole to HCMV DNA packaged into capsids inhibits structural changes to capsids during maturation to virions in the cytoplasm, which leads to capsid destruction in the cytoplasm.

We found that Ridinilazole had no obvious antiviral effect on either HSV-1 or HSV-2. Our observations are consistent with previous published observations that a high concentration of Hoechst 33342 had no antiviral effect on HSV-1 replication [4] and our own as yet unpublished observations that a high concentration of RO-90-7501 had no antiviral effect on HSV-1 replication (data not shown). It remains unclear why Ridinilazole had antiviral effects against HCMV, but not HSV. We speculate that this may be due to the fewer number of putative compound binding sites in HSV genomes, compared to HCMV genomes. This infers that Ridinilazole may not have a broad antiviral effect against a range of DNA viruses and that the number of putative compound binding sites should be considered using our bioinformatic approach before testing for antiviral activity of Ridinilazole (or other bisbenzimides) on other DNA viruses.

We also note that the bisbenzimide compounds examined here are unlikely to be DNA modifying, as they do not intercalate into DNA [1,3,12], unlike DNA-modifying compounds such as cisplatin. This is supported by the previous observation that Ridinilazole had anti-proliferative effects in cisplatin-resistant cell lines [13]. It remains a possibility that mutation of the HCMV genome may result in drug resistance mutations allowing escape from the antiviral activity of Ridinilazole. However, we have as yet been unable to isolate HCMV viruses resistant to either RO-90-7501 or Ridinilazole (data not shown). These observations are likely due to the very large number of putative bisbenzimide binding sites present in HCMV virus genomes. Regardless, the apparent inability of HCMV to escape the antiviral activity of bisbenzimide compounds may be helpful in future.

A potential drawback to the use of Ridinilazole as a future antiviral agent is our observation that the antiviral activity of Ridinilazole on HCMV was reversible. This is likely due to reversible binding of the compound to DNA. We and others have observed that the current frontline anti-HCMV drug Foscarnet has reversible anti-HCMV activity [28,29]. Thus, reversible antiviral activity would not itself preclude future clinical use of Ridinilazole. However, careful clinical studies would be required to understand Ridinilazole dosing to provide an antiviral effect.

A further potential drawback to the future clinical use of Ridinilazole as an antiviral agent is that this compound is not readily orally bioavailable [14,16]. However, this does not immediately prevent its use as an antiviral compound. It is possible that this compound could be administered by injection, similar to other antiviral agents. Plus, there may be *ex vivo* usage; for example, treatment of HCMV-infected tissue for transplant. Looking to the future, modifications to the structure of Ridinilazole could be made that would allow the compound to obey the Lipinski rules of oral bioavailability. Our previous examination of RO-90-7501 and MRT00210423 demonstrates that additions can be made to the termini of bisbenzimide compounds while retaining anti-HCMV activity [5]. Thus, the termini of Ridinilazole could be convenient sites for modification of the compound so that it will obey the Lipinski rules.

A major driver of investigating a possible antiviral activity of Ridinilazole was that there is ample data that this compound can be administered in human clinical trials, with no obvious adverse effects [14,16]. Although these trials were successful in demonstrating an antibacterial effect *in vivo* [14,16], Ridinilazole was originally developed as an anticancer agent [11]. However, it is interesting to speculate why we did not observe the previously described *in vitro* anti-cell division activity of Ridinilazole [13]. This may be due to differing experimental conditions and/or that the compound acts differently on some transformed cancer cells compared to unmodified primary human cell cultures. Regardless, as noted above, so far administration of Ridinilazole has had no obvious adverse effects in humans [14,16].

Finally, development of Ridinilazole may be useful beyond inhibition of HCMV replication. As we and others have previously demonstrated, bisbenzimide compounds are efficacious inhibitors of poxvirus replication [4,6]. Therefore, testing of Ridinilazole against poxviruses, including mpox, may indicate that Ridinilazole is an efficacious inhibitor of those viruses. Plus, a recent report has indicated that Hoechst 33342 was an inhibitor of African swine fever virus (ASFV) [30]. Therefore, testing of Ridinilazole should be extended beyond human viruses into viruses which cause significant disease in animals, such as ASFV.

## Abbreviations

ASFV: African swine fever virus
DMEM: Dulbecco’s modified eagle’s medium
HCMV: human cytomegalovirus
HFF: human foreskin fibroblast
HSV: herpes simplex virus
MDCK: Madin-Darby canine kidney
MPXV: monkeypox virus
MTT: 3-(4,5-dimethylthiazol-2-yl)-2,5-diphenyltetrazolium bromide
SPB: Sorensen’s phosphate buffer
TEM: transmission electron microscopy
TPCK: L-1-tosylamido-2-phenylethyl chloromethyl ketone
VV: vaccinia virus
